# Supplementary figures and images for: Anti-inflammatory activity of soluble chito-oligosaccharides (CHOS) on VitD3-induced human THP-1 monocytes
Source: PLoS One. 2021 Feb 3;16(2):e0246381. doi: 10.1371/journal.pone.0246381 (PMC7857634; doi:10.1371/journal.pone.0246381)

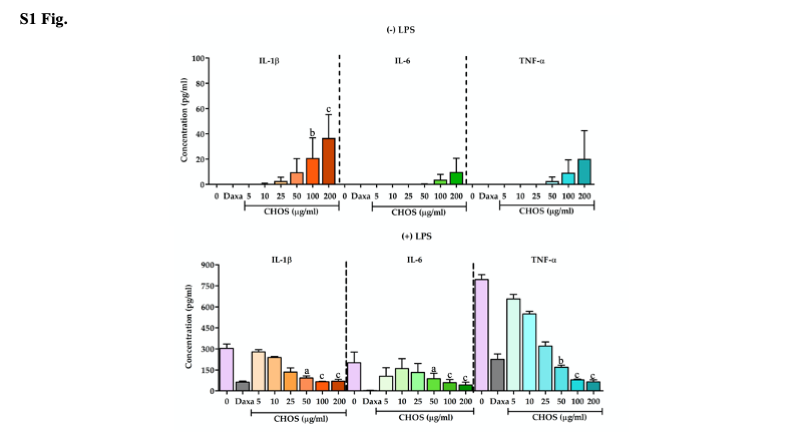

Supplement: S1 Fig — The results of Fig 6 are plotted with a different Y-axis scale, to highlight the values of samples without LPS treatment. (TIF) [file pone.0246381.s001.tif]

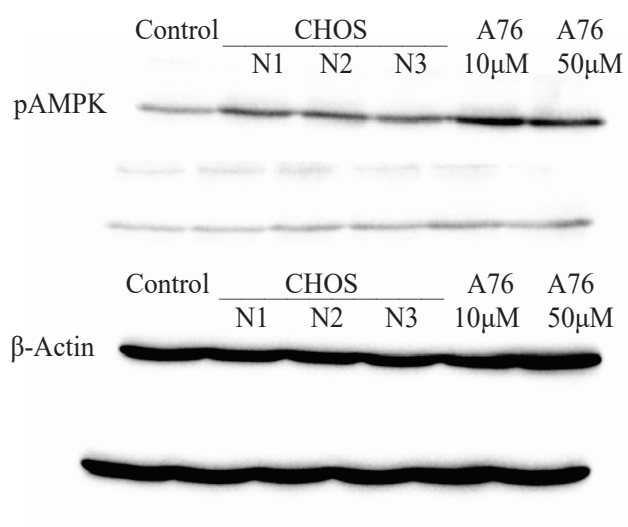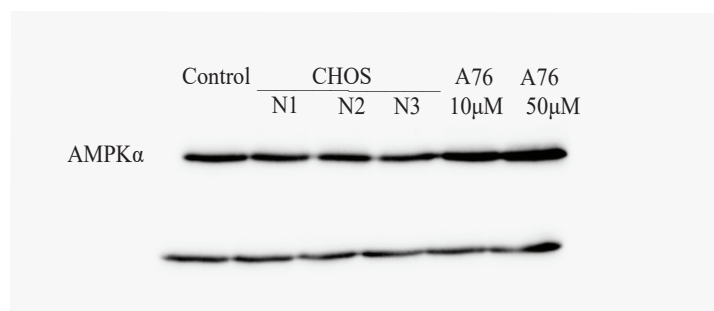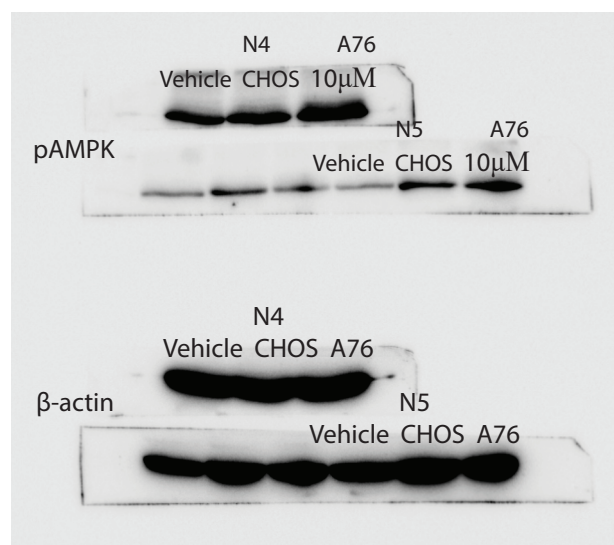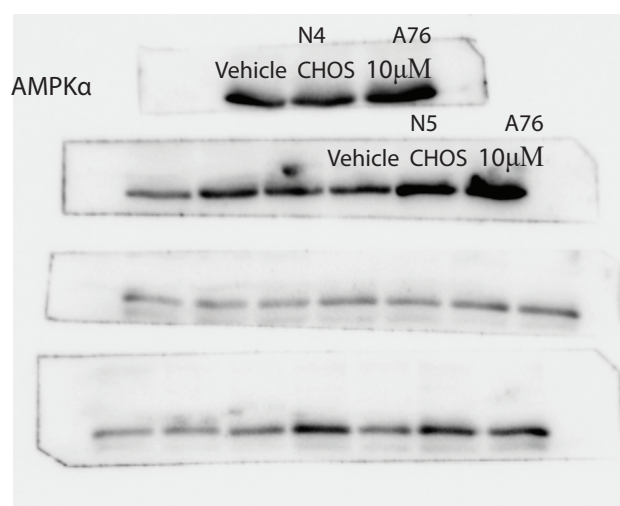

Supplement: S1 File — (PDF) [file pone.0246381.s003.pdf]
