# Supplementary material for: Anti-inflammatory activity of soluble chito-oligosaccharides (CHOS) on VitD3-induced human THP-1 monocytes
Source: PLoS One. 2021 Feb 3;16(2):e0246381. doi: 10.1371/journal.pone.0246381 (PMC7857634; doi:10.1371/journal.pone.0246381)
Supplement: S1 Table — (PDF) [file pone.0246381.s002.pdf]

Data of Statistical Analysis

Statistical Analysis of Fig. 3

Conditions	Sample Group (mean±SD, n=6)		
	**Cont. (-LPS)**	**0 µg/mL CHOS (+LPS)**	**100 µg/mL CHOS (+LPS)**
PMA	2.93 (±3.21)	123.59 (±5.79)	97.50 (±5.40)
p-value		*< 0.001*[a]	*< 0.001*[a], *< 0.001*[a*]
VitD3	0.27 (±0.60)	237.582 (±9.27)	41.74 (±4.90)
p-value		*< 0.001*[a]	*< 0.001*[a], *< 0.001*[a*]

a and a indicated significant; a*: versus control (-LPS); a: versus 0 µg/mL CHOS, p < 0.001*

Statistical Analysis of Fig. 4

Conditions	Sample Group (medians; 25%, 75%; n=6)			
	**0 h**	**48 h**	**72 h**	**96 h**
% CD14 of PMA	0.84 (0, 2.11)	10.68 (9.48, 12.16)	18.03 (17.36, 19.20)	16.56 (15.6, 17.88)
p-value		*0.424*	*< 0.001*[a]	*0.004*[b]
% PI of PMA	1.50 (0.89, 1.742)	23.85 (23.37, 25.88)	10.51 (9.93, 11.06)	23.87 (23.25, 25.52)
p-value		*< 0.001*[a]	*0.425*	*< 0.001*[a]
% CD14 of VitD3	0.74 (0, 1.85)	80.74 (77.22, 81.39)	0.15 (0, 0.35)	95.16 (94.01, 95.85)
p-value		*0.424*	*< 0.001*[a]	*< 0.001*[a]
% PI of VitD3	0.68 (0.13, 1.11)	0.13 (0, 0.30)	0.15 (0, 0.35)	0 (0.21, 0.53)
p-value		*0.071*	*0.146*	*0.427*

p-value versus control (0 h); a = sig. < 0.001; b = sig. < 0.01

Statistical Analysis of Fig. 5

**Conditions**	**Sample Groups** (medians; 25%, 75%; n=9)							
	**0 µg/mL**	**5 µg/mL CHOS**	**10 µg/mL CHOS**	**25 µg/mL CHOS**	**50 µg/mL CHOS**	**100 µg/mL CHOS**	**200 µg/mL CHOS**	**100 ng/mL LPS**
% Cell viability	102.3 (98.90, 102.7)	103.6 (97.84, 105.1)	102.9 (101.3, 104.4)	106.2 (105.5, 107.4)	101.8 (92.13, 105.2)	102.6 (96.95, 106.5)	104.8 (100.5, 109.2)	98.54 (95.14, 99.77)
***p*-value**		*> 0.999*	*> 0.999*	*> 0.999*	*0.123*	*> 0.999*	*> 0.999*	*> 0.999*

p-value versus control (0 µg/mL)

Statistical Analysis of Fig. 6

**Conditions**	**Sample Groups** (medians; 25%, 75%; n=6)							
	**Control**	**0.5 µM Daxa**	**5 µg/mL CHOS**	**10 µg/mL CHOS**	**25 µg/mL CHOS**	**50 µg/mL CHOS**	**100 µg/mL CHOS**	**200 µg/mL CHOS**
Non LPS-stimulated (IL-1β)	0.00	0.00	0.00	0 (0, 0.46)	2.32<br>(0, 5.56)	8.11<br>(0, 20.44)	21.46<br>(5.42, 36.08)	36.57<br>(19.08, 54.71)
***p*-value**	-	*> 0.999*	*> 0.999*	> 0.999	*> 0.999*	*0.575*	*0.005*[b]	*> 0.001*[a]
Non LPS-stimulated (IL-6)	0.00	0.00	0.00	0.00	0.00	0<br>(0, 0.5)	3.45<br>(0, 7.58)	9.22<br>(0, 19.79)
***p*-value**	-	*> 0.999*	*> 0.999*	*> 0.999*	*> 0.999*	*> 0.999*	*0.176*	*0.081*
Non LPS-stimulated (TNF-α)	0.00	0.00	0.00	0.00	0.00	1.59<br>(0, 5.67)	7.83<br>(0, 18.98)	19.45<br>(0, 40.32)
***p*-value**	-	*> 0.999*	*> 0.999*	*> 0.999*	*> 0.999*	*0.395*	*0.205*	*0.099*
LPS-stimulated (IL-1β)	301.2<br>(274.4, 334.6)	62.57<br>(54.8, 69.28)	274.8<br>(264.6, 295.8)	237.2<br>(231.7, 245.9)	135.5<br>(105.2, 164.4)	90.75<br>(79.28, 107.0)	65.74<br>(63.73, 68.37)	67.46<br>(58.02, 80.44)
***p*-value**	-	*< 0.001*[a]	*> 0.999*	*> 0.999*	*0.236*	*0.038*[c]	*< 0.001*[a]	*< 0.001*[a]
LPS-stimulated (IL-6)	266.3<br>(262.3, 270.2)	2.25<br>(0, 4.67)	211.2<br>(191.3, 190.6)	173.6<br>(145.5, 190.6)	112.3<br>(102.1, 123.4)	77.72<br>(74.35, 81.3)	53.79<br>(47.35, 62.12)	50.15<br>(42.15, 61.83)
***p*-value**	-	*< 0.001*[a]	*> 0.999*	*> 0.999*	*0.182*	*0.021*[c]	*< 0.001*[a]	*< 0.001*[a]
LPS-stimulated (TNF-α)	797<br>(758, 827)	223<br>(190, 261)	674<br>(618, 678)	555<br>(535, 561)	300<br>(313, 334)	172<br>(152, 182)	78.7<br>(75.2, 82.4)	65.1<br>(49.7, 78.5)
***p*-value**	-	*0.02*[c]	*> 0.999*	*0.963*	*0.182*	*0.002*[b]	*< 0.001*[a]	*< 0.001*[a]
LPS-stimulated (n=3) (IL-1b; Washing CHOS)	45.62<br>(42.76, 51.06)	-	-	-	-	21.24<br>(17.22, 34.19)	12.76<br>(10.44, 16.24)	-
***p*-value**	-					*0.359*	*0.015*[c]	

p-value versus control; a = sig. < 0.001; b = sig. < 0.01; c =sig. <0.05

Statistical Analysis of Fig. 7

**Conditions**	**Sample Groups** (medians; 25%, 75%; n=6)			
	**Control**	**CHOS**	**VitD3 alone**	**VitD3 (CHOS)**
Non LPS-stimulated (% CD14)	0.87 (0.78, 2.46)	2.12 (1.60, 2.53)	81.7 (80.86.3, 83.35)	90.46 (90.17, 95.1)
p-value		*> 0.999*	*0.054*	*< 0.001*[a]*, 0.85*[ns]
LPS-stimulated (% CD14)	1.37 (0.78, 1.48)	2.41 (1.38, 2.56)	91.37 (88.14.3, 95.15)	94.36 (93.6, 95.48)
p-value		*> 0.999*	*0.012*[c]	*0.001*[b]*, > 0.999*[ns]

p-value versus control; a = sig. < 0.001); b = sig. < 0.01; c = sig. < 0.05; ns = not significant when versus VitD3 alone
