# Supplementary material for: Anti-inflammatory activity of soluble chito-oligosaccharides (CHOS) on VitD3-induced human THP-1 monocytes
Source: PLoS One. 2021 Feb 3;16(2):e0246381. doi: 10.1371/journal.pone.0246381 (PMC7857634; doi:10.1371/journal.pone.0246381)
Supplement: Attachment — Submitted filename: Point-by-point responses 2nd revision-02122020.docx [file pone.0246381.s005.docx]

**Second (minor) revision of “Anti-inflammatory activity of soluble chito-oligosaccharides (CHOS) on VitD3-induced human THP-1 monocytes” (PONE-D-20-27129R1) based on comments received from the Editor.**

**Detailed description of the revision:**

Study strength and limitation has been added before the conclusion section of the second revised manuscript.

1- Avoid using "We, our". Use impersonal phrasing throughout the text

**Response: Done. "**We" and "our" have been removed from the manuscript.

2- Abbreviations MUST be stated in full acronym before being abbreviated throughout the MS

**Response: Checked.**

3- Don't use abbreviations in keywords. limit them to 5 to 6 keywords

**Response: Done.** The number of keywords has been limited to six and none of these are abbreviations..

4- Method performance and optimization should be provided in section "Analysis of CHOS by MS"

**Response:** There is not really anything to report here. These were just typical, and very commonly used, MALDI experiments, using standard conditions, which are described in the manuscript. There was no optimization other than what one normally does, namely moving the laser over the spots until one obtains the best possible spectra; since this is inherent for a MALDI experiment, it would be strange to report this. The choice of the matrix was based on the cited paper (Bahrke et al., 2020). Furthermore, we do not really see how one could report the “performance” of these experiments. We do not understand which performance parameters one could use (resolution? – resolution is massively sufficient) and why these would be relevant. Importantly, this type of MALDI analysis primarily provides quantitative information (i.e., which products are there); quantitative interpretation should be done with great care and has limited reliability. Therefore, quantitative statements regarding the results in Table 1 are made with great care. To make this even clearer we deleted the words “estimates using” from the footnote of Table 1, since these words may suggest that we did calculations, which we, obviously, did not.

Based on these considerations, we respectfully argue that there is no need for further changes regarding the MALDI-MS part of the manuscript.

5- The P letter for statistical analysis should be uppercase-italic face letter. Hz should be italized as well

**Response: Done.** Thank you very much for pointing out these mistakes.

6-Study strength and limitations should be separated from the section "Concluding remarks". This means line 536-546 should be under heading "Study strength and limitations". The rest should be under conclusion

**Response: Done.**

7- If for any reason, the authors' can't do the experimental work suggested by the diligent reviewers, this at least MUST be acknowledged under study limitation

**Response: Done.** We have acknowledged this study limitation in the "Study strength and limitations" section of the revised manuscript by adding the following text:

*"Moreover, transcriptomic analyses, which could help elucidate the mechanism of action of CHOS have not been done in this study. Such deeper analyses, including in vivo analysis in humans (clinical trials), are of great interest and needed to eventually bring CHOS to the clinic.”*

8- Proofread the text for grammar and syntax errors

**Response: Done.** We have carefully proofread the text and several minor corrections have been made.
